# Predictors of smoking cessation behavior among Bangladeshi adults: findings from ITC Bangladesh survey

**DOI:** 10.1186/s12971-015-0050-y

**Published:** 2015-08-11

**Authors:** Abu S. Abdullah, Pete Driezen, Anne C. K. Quah, Nigar Nargis, Geoffrey T. Fong

**Affiliations:** Department of General Internal Medicine, Boston Medical Center, Boston University School of Medicine, 801 Massachusetts Avenue, 2nd Floor (MISU), Boston, Massachusetts 02118 USA; Global Health Program, Duke Kunshan University, Kunshan, Jiangsu Province 215347 China; Duke Global Health Institute, Duke University, Durham, NC 27710 USA; Department of Psychology, University of Waterloo, Ontario, Canada; Department of Economics, University of Dhaka, Dhaka, Bangladesh; Ontario Institute for Cancer Research, Toronto, Ontario Canada

**Keywords:** Smoking, Quit attempt, Smoking cessation, Bangladeshi adults

## Abstract

**Background:**

Research findings on the predictors of smoking cessation behavior identified in Western countries may not be generalizable to smokers in the Southeast Asian countries (i.e., Bangladesh). This study examined the factors associated with smoking cessation behavior (quit attempts and smoking cessation) among a representative sample of Bangladeshi adults.

**Methods:**

Data from Wave 1 (2009) and Wave 2 (2010) of the International Tobacco Control (ITC) Survey in Bangladesh, a face-to-face survey of adult smokers, were analysed. Households were sampled using a stratified multistage design and interviewed using a structured questionnaire. Respondents included in the study are 1,861 adult daily smokers (cigarette only or dual use of cigarette and bidi) in the Wave 1 survey who completed the Wave 2 follow up.

**Results:**

Of the smokers (*N* = 1,861), 98 % were male, 18 % illiterate, 78 % married and 42 % were aged 40 or above; 89 % were cigarette smokers and 11 % were dual users (cigarette & bidi). Overall, 21.8 % of the baseline smokers made quit attempts (that is, making at least one quit attempt that lasted for at least 24 hours) during the 11- to 12-month interval between Waves 1 and 2 with only 4.1 % quitting successfully (that is, smokers who had stopped smoking for at least 6 months at the time of the Wave 2 survey). Significant predictors of attempts to quit included: residing areas outside Dhaka (*OR* = 3.41), being aged 40 or older (*OR* = 1.53), having a monthly income of above BDT10,000 (US$126) versus below BDT 5,000 (US$63) (*OR* = 1.57), intending to quit sometime in the future (*OR* = 1.73). Respondents not working indoors/outside the home were less likely to have made a quit attempt than those with no workplace restrictions on smoking (*OR* = 0.62). Predictors of successful smoking cessation included: being aged 40 or older (*OR* = 3.11), perceiving self-rated health as good or excellent (*OR* = 2.40), and an increased level of self-efficacy (*OR* = 1.75). Smokers who made a quit attempt not so recently (6 months ago or earlier) were less likely to quit than those who made a more recent (in last 6 months) quit attempt (*OR* = 0.23).

**Conclusion:**

Among Bangladeshi smokers, different factors were associated with quit attempt or successful cessation. Population based smoking cessation programs should take these factors into consideration in the design of smoking cessation interventions. At the same time, measures are necessary to encourage more smokers to make quit attempts.

## Introduction

Tobacco use is the leading cause of preventable death and disease worldwide and is estimated to kill more than 5 million people each year [1]. Public health strategies to combat tobacco-induced illnesses have aimed at reducing uptake and promoting smoking cessation. Given the limitations of population-based programs to reduce smoking initiation among the public [2], the need to provide cessation support to those who continue to smoke is substantial. Therefore, identification of factors that could facilitate cessation is important in the design of evidence-based smoking cessation interventions.

With a population of 150 million, Bangladesh is one of the top ten countries in the world having a high smoking prevalence, where over 22 million adults smoke [3]. The overall smoking (cigarettes, bidis, and hookah) prevalence has increased in Bangladesh from 20.9 % in 2004 to 22.0 % in 2010 [4]. Studies also identified a 9-percentage point increase of smoking-attributable deaths among Bangladeshi adults; from 16 % in 2004 [5] to 25 % in 2010 [6]. This high rate of tobacco-attributable mortality underscores the rapidly growing health and economic burden of tobacco use in Bangladesh. To address this growing epidemic of tobacco-induced deaths, there is an urgent need to reduce tobacco use in Bangladesh which will require curtailing initiation of tobacco use and promotion of smoking cessation.

Previous research [7–10] has identified factors associated with smoking cessation, including low nicotine dependence, male gender, higher educational attainment, being married, being older, consuming fewer cigarettes per day, and not having other smokers in the household. However, little is known about the factors that are associated with smoking cessation among the Bangladeshi population. The identification of Bangladesh-specific factors is needed to assess the need for and nature of smoking cessation services that would be appropriate for Bangladesh and other developing countries with similar socioeconomic characteristics.

The aim of this study is to examine the factors associated with smoking cessation behavior (that is, quit attempts and smoking cessation) among a nationally representative sample of Bangladeshi adults.

## Methods

### Sample

The data for this study come from the first two waves of the International Tobacco Control (ITC) Policy Evaluation Bangladesh Survey. The ITC Bangladesh Survey is a prospective cohort survey of a nationally representative sample of adult smokers and non-smokers (aged 15 and older) conducted in all six administrative divisions of Bangladesh [11, 12]. In total, 1,792 cigarette smokers and 229 dual users (defined as smokers who smoke both cigarettes and bidis) were recruited in Wave 1 from the non-tribal and non-slum areas of Bangladesh. Of these, 1,656 cigarette smokers (92 %) and 205 dual users (90 %) were successfully followed in Wave 2. The analysis reported here is based only on the sample of smokers recruited in Wave 1 and retained in Wave 2. Data were collected using face-to-face interviews and sampling weights were computed so that results are representative of the population of adult Bangladeshi smokers. The details of the ITC Bangladesh Survey are described elsewhere [4, 12, 13].

### Measures

Attempts to quit smoking (i.e. quit attempts) and successful cessation formed the primary outcome measures used in this study. Quit attempts were defined as making any serious attempt to quit smoking, between the waves, that lasted for at least 24 hours. Successful cessation in Wave 2 was defined as making a quit attempt between the waves and not smoking for six months or longer as reported by the respondents.

#### Predictor variables

The ITC Bangladesh Survey measures a wide range of domains related to tobacco use and tobacco control. Details of the questionnaire are described elsewhere [12] but relevant domains include socio-demographic characteristics as well as behavioural, cognitive, attitudinal, environmental and motivational measures. All independent variables were measured in Wave 1 and used to predict cessation attempts and successful cessation in Wave 2.

#### Socio-demographic measures

Respondents’ socio-demographic backgrounds were characterized using sex, age, religion (Muslim vs. non-Muslim), residence (Dhaka vs. areas outside Dhaka) and marital status (married vs. not). The ITC Bangladesh Survey also assesses respondents’ education (illiterate, 1--8 years and 9 years or more), monthly household income (<5,000 BDT, 5,000--10,000 BDT, > 10,000 BDT and not reported) (exchange rate: 1US$ = 79BDT), and the number of smokers living in each respondent’s home.

#### Smoking behaviors

Smokers were classified on the basis of whether they smoked on a daily or non-daily basis. In addition, smokers reported their usual daily consumption of cigarettes and bidis. Typical consumption was treated as a continuous variable; for cigarette smokers, total consumption was defined using the number of cigarettes smoked per day. For dual users, total consumption was defined as the number of cigarettes *and* then number of bidis smoked per day. Smokers were also asked how recently they tried to quit smoking (never tried, within the last six months and six months ago or longer). Finally, smokers reported the age at which they started smoking. This information was used to compute the number of years each respondent smoked, using respondent’s age at recruitment into the ITC Bangladesh Survey.

#### Beliefs

This study assessed respondents’ self-reported health (average or poor vs. good or excellent), their level of addiction to cigarettes (not addicted, somewhat addicted or very addicted), intentions to quit smoking (no plans, sometime in the future or within the next six months) and beliefs about their confidence (self-efficacy) to quit (measured on a five point scale ranging from not at all sure to extremely sure). With the exception of self-efficacy, all belief variables were treated as categorical measures in the analysis.

#### Environmental factors

Smokers’ surrounding environments were assessed on the basis of the number of smokers living in their homes (1, 2 and 3 or more) and whether they received any support to quit smoking (defined as advice, information or referral to quit given by a physician or health care provider). Respondents were also asked about whether they themselves had implemented any smoking restrictions in their homes (no restrictions, partial restrictions or complete restrictions). In addition, smokers who worked outside the home were asked about workplace smoking restrictions; smokers were classified as not working outside the home, not working indoors, having no or only partial workplace restrictions and having complete workplace smoking bans.

#### Motivational factors

Smokers’ motivation to quit smoking was assessed by measuring (a) their overall opinion towards cigarette smoking, (b) their overall opinion towards bidi smoking, (c) their expectations of future health effects, if they quit smoking (outcome expectancy), (d) their worry about the health consequences of smoking, and (e) whether they have favourable attitudes toward smoking. Opinions toward smoking were classified as good or neutral, bad, and very bad. Outcome expectancy, worries about health and favourable attitudes toward smoking were measured as continuous variables. Outcome expectancy was assessed using the question “How much do you think you would benefit from health and other gains if you were to quit smoking cigarettes permanently in the next 6 months?” Responses ranged from 1 = “not at all” to 5 = “extremely”. Worries about health were defined using the average of two measures: “How worried are you, if at all, that smoking cigarettes will damage your health in the future?” and “To what extent, if at all, has smoking cigarettes lowered your quality of life?” Each of these measures was assessed using a four-point scale (worry: 1 = “not at all worried”, 2 = “a little worried”, 3 = “moderately worried”, and 4 = “very worried”; quality of life: 1 = “not at all”, 2 = “just a little”, 3 = “a fair amount”, 4 = “a great deal”). Favorable attitude toward smoking was also assessed as the average of two measures: (a) “enjoy smoking too much to give it up” and (b) “smoking cigarettes is an important part of your life”. Each of these measures was assessed using a five-point Likert scale ranging from 1 = “strongly agree” to 5 = “strongly disagree”. All of the continuous motivation measures were coded so that higher scores reflected greater amounts of each measure.

#### Reasons to quit

Reasons to quit smoking included smokers’ concern about the effect of cigarette smoke on non-smokers, believing that Bangladeshi society disapproves of smoking, the price of cigarettes, workplace smoking restrictions, smoking restrictions in public places, free or low-cost smoking cessation medications, advertisements about the health risks of smoking, warning labels on cigarette packs, setting an example for children, friends and family disapprove of smoking, and the rising costs of essentials including food or fuel. Each measure was classified on the basis of whether or not it was a reason to quit smoking.

### Statistical Analysis

Descriptive statistics appropriate for complex survey data were used to estimate the prevalence of cessation attempts and successful cessation by Wave 2. Bivariate associations between quit status, defined as no attempts to quit, tried but unsuccessful and successful cessation, and each of the categorical measures were tested using the Rao-Scott χ^2^ statistic. Mean levels of the continuous predictors were estimated for each quit status category. Differences between categories were tested using a univariate linear regression model, accounting for the complex survey design.

Binary logistic regression models were then used to examine associations between independent predictors and (1) attempts to quit and (2) successful cessation.

The first set of models examining predictors of quit attempts were based on all smokers. Following Borland, et al., [14], smokers who tried to quit but were not successful and smokers who quit successfully were classified as having tried to quit. These models estimated the odds of making an attempt to quit (vs. not making an attempt). Models predicting successful cessation were based on only those smokers who tried to quit to estimate the odds of successful cessation (vs. trying, but failing, to quit).

Regression models were built using a backward selection procedure. All covariates were entered into a preliminary model while socio-demographic covariates were forced into each model. Then, each behavioural, belief, environmental and motivational covariate was removed from the preliminary model, one variable at a time. In each step, the Akaike information criterion (AIC) was computed and the best fitting sub-model was selected (i.e., the sub-model having the smallest AIC statistic after removing a covariate). The procedure was repeated until removal of variables no longer improved model fit (i.e., no sub-model resulted in a smaller value for the AIC statistic). The final selected model was then re-fit using only the selected covariates in order to use as many observations as possible to predict each of the cessation outcomes. The selection procedure was conducted using logistic regression procedures appropriate for complex survey data in order to account for the multi-stage sampling design. All results presented here are weighted using a longitudinal weight that adjusts for attrition in order to represent the population of adult smokers aged 15 and older in Bangladesh. The analysis was conducted using SAS version 9.4.

## Results

Of 1,861 daily smokers present in both Waves 1 and 2 (Table 1), the majority (85 %) were recruited from non-tribal areas outside Dhaka. The vast majority of smokers were male (98 %), married (78 %), Muslim (87 %), smoked cigarettes exclusively (89 %), and had monthly household incomes of more than 5,000 BDT (1US$ = 79 BDT) (74.7 %). Almost three quarters of selected smokers came from homes having two or more smokers. Overall, 4 % of adult smokers quit smoking for at least six months in 2010 while another 22 % tried to quit but were not successful.

**Table 1:** Sample characteristics of smokers recruited in Wave 1 and followed to Wave 2 (unweighted; *n* = 1861)

	%	(Freq.)
Area		
Dhaka	15.1	(281)
Areas outside Dhaka	84.9	(1580)
Sex		
Male	98.1	(1826)
Female	1.9	(35)
Age group		
Younger than 40	57.8	(1076)
40 or older	42.2	(785)
Marital status		
No married	22.2	(413)
Married	77.8	(1444)
Religion		
Non-Muslim	12.6	(234)
Muslim	87.4	(1626)
Education		
Illiterate	18.6	(346)
1 to 8 years	55.3	(1026)
9+ years	26.1	(484)
Income		
<5,000 BDT	15.8	(294)
5,000 to 10,000 BDT	42.7	(794)
>10,000 BDT	32.2	(599)
Not reported	9.3	(174)
Total smokers in home		
1	25.7	(478)
2	52.8	(982)
3 or more	21.5	(401)
Type of smoker		
Exclusively cigarettes	89.0	(1656)
Dual user	11.0	(205)
Mean daily amount smoked (SD)		
Exclusively cigarettes	9.60	(±6.16)
Dual user	13.32	(±8.82)
Quit status		
Did not try to quit	74.1	(1379)
Tried unsuccessfully	21.8	(406)
Quit successfully	4.1	(76)

*BDT* = Bangladeshi Taka; 1US$ = 79BDT

Bivariate associations between demographic variables and Wave 2 quit status (quit attempts and successful quitting) showed that older age and area of residence were associated with quit status. Specifically, older (aged 40 or above) smokers were significantly more likely to try to quit (25.2 %) or quit successfully (6.4 %) than younger (aged 15–39) smokers (20.5 % and 2.7 %, respectively) (χ^2^ = 15.10; p < 0.001). Subjects recruited from outside of Dhaka were significantly more likely to try to quit (22.9 %) or quit successfully (4.4 %) than Dhaka (13.7 % and 1.3 %, respectively) (χ^2^ = 8.86; p = 0.012). (Data not shown)

Tables 2 and 3 examine bivariate associations between Wave 2 quit status (quit attempts and successful quitting) and behavioural, belief, environmental and motivational factors. Many of these factors were not significantly associated with quit status. However, quit recency, perceived addiction, quit intentions and workplace smoking restrictions were significantly associated with quit status (Table 2). In addition, cigarette consumption (mean numbers smoked daily) and attitudes toward smoking differed by quit status (Table 3). A greater percentage of smokers who made previous attempts to quit had tried again by wave 2 compared to smokers who never tried to quit. Specifically, almost 30 % of smokers who ever tried to quit in Wave 1 reported making additional attempts to quit in Wave 2. Another 6.1 % of smokers who, in Wave 1, tried to quit within the last six months managed to quit successfully by Wave 2 (Table 2). A larger percentage of smokers who planned to quit sometime in the future in Wave 1 reported making additional attempts to quit by wave 2, compared to smokers who never tried to quit in Wave 1 (29.3 % vs 17.5 %, respectively). Smokers having more definite plans to quit in Wave 1 were more likely to have quit successfully by Wave 2: 7.7 % of smokers who planned to quit within the next six months quit successfully by Wave 2 compared to less than 4 % of smokers who had no plans or non-definite plans to quit in Wave 1 (Table 2). Other factors significantly related to quit status included daily cigarette consumption and holding favorable attitudes toward smoking (Table 3).

**Table 2:** Behavioural, belief, environmental and motivational factors associated with Wave 2 quit status, ITC Bangladesh (weighted results)

	Wave 2 quit status								
	Did not try to quit		Tried, unsuccessful		Quit successfully				
	Freq.	%	Freq.	%	Freq.	%	Rao-Scott χ^2^	DF	p value
Smoking behaviours									
Type of smoker									
Exclusively cigarettes	1220	74.6	368	21.6	68	3.8	0.40	2	0.818
Dual user	159	77.6	38	19.6	8	2.8			
Any tobacco use									
Cigarettes & other smoked or smokeless	161	77.8	38	19.4	8	2.8	1.80	4	0.772
Cigarettes & smokeless	227	73.2	80	21.6	20	5.2			
Exclusively cigarettes	991	75.0	288	21.6	48	3.4			
Cigarettes/day									
<= 10/day	945	74.2	278	21.8	59	4.0	2.43	4	0.658
11-20/day	337	75.2	109	21.7	14	3.1			
21+/day	66	82.9	16	16.4	2	0.7			
Quit recency									
Never tried to quit	896	78.6	214	17.9	42	3.5	25.67	4	<0.001
Recently (in last 6 months)	161	66.7	67	27.2	15	6.1			
Not so recently (6 months ago or earlier)	293	68.9	121	28.6	16	2.5			
Quit beliefs									
Self-rated health									
Poor/average health	633	72.2	202	24.3	35	3.5	5.97	2	0.051
Good/excellent health	744	77.4	204	18.7	41	3.8			
Perceived addiction									
Not addicted	128	65.4	42	28.4	12	6.2	8.46	4	0.076
Somewhat addicted	833	74.7	254	21.8	46	3.5			
Very addicted	408	78.4	107	18.4	17	3.2			
Quit intentions									
No plan to quit	682	79.8	169	17.5	25	2.6	33.34	4	<0.001
Sometime in the future	437	67.1	181	29.3	31	3.6			
Within the next 6 months	134	70.8	40	21.5	12	7.7			
Environmental factors									
Total smokers in home									
1	365	75.7	91	20.5	22	3.8	1.45	4	0.835
2	724	75.1	217	21.0	41	3.9			
3 or more	290	73.6	98	23.5	13	2.9			
Cessation support									
No advice	1213	75.6	354	20.9	62	3.5	2.55	2	0.279
Advice/information/referral to quit	166	69.5	52	25.4	14	5.1			
Workplace smoking restrictions									
No restrictions	205	74.5	70	22.2	8	3.3	1.95	4	0.745
Partial/complete restrictions	181	72.0	64	24.9	9	3.1			
Does not work indoors/does not work	964	75.7	258	20.3	59	4.0			
Smoking restrictions in the home									
No restrictions	527	75.6	163	21.2	28	3.1	4.14	4	0.388
Partial restrictions	213	80.7	54	17.0	8	2.4			
Completely banned	610	72.0	185	23.6	38	4.4			
Motivational factors									
Overall opinion towards cigarette smoking									
Good/neutral	66	77.7	13	17.4	5	4.9	1.31	4	0.860
Bad	1019	74.9	305	21.7	50	3.4			
Very bad	286	74.2	87	21.5	21	4.3			
Overall opinion towards bidi smoking									
Good/neutral	35	85.8	7	12.5	1	1.7	4.73	4	0.316
Bad	875	76.1	244	20.4	42	3.5			
Very bad	462	72.1	153	23.7	33	4.2

**Table 3:** Mean levels of behavioural, belief and motivational factors by Wave 2 quit status (weighted)

	Wave 2 quit status									
	Did not try to quit		Tried, unsuccessful		Quit successfully					
	N	Mean	N	Mean	N	Mean	F Test		DF	p value
Behavioural factors										
Cigarettes/day										
Mean cig/day	1348	10.08	403	9.52	75	7.65	8.20	2,	32	0.001
Mean no. years smoked										
Mean no. years smoked	1379	18.15	406	20.44	76	23.06	2.29	2,	32	0.117
Quit beliefs										
Self-efficacy										
Mean self-efficacy	1363	2.14	404	2.16	76	2.37	0.72	2,	32	0.496
Motivational factors										
Outcome expectancy										
Mean	1345	2.86	390	2.97	69	2.85	1.31	2,	32	0.284
Worries about health										
Mean	1340	2.18	389	2.17	73	2.30	1.42	2,	32	0.256
Favourable attitude towards cigarette smoking										
Mean	1330	3.08	393	2.97	70	2.86	7.05	2,	32	0.003

To better understand the predictors of (a) attempts to quit smoking and (b) successful smoking cessation among Bangladeshi smokers, weighted multi-variable logistic regression was used to model the independent predictors of each outcome controlling for socio-demographic covariates. As shown in Table 4, significant predictors of attempts to quit included: residing outside of Dhaka (OR = 3.41), being aged 40 or older (OR = 1.53), having a monthly income of above BDT 10,000 versus below BDT 5,000 (OR = 1.57), having an intention to quit sometime in the future versus having no plans to quit (OR = 1.73). Respondents not working indoors/outside the home were less likely to have made a quit attempt than those with no workplace restrictions on smoking (OR = 0.62). As shown in Table 5, predictors of successful smoking cessation included: being aged 40 or older (OR = 3.11), perceiving self-rated health as good or excellent (OR = 2.40), and an increased level of self-efficacy (OR = 1.75), where a one point increase in smoker’s self-efficacy increased the odds of successful cessation by 80 %. Smokers who made a quit attempt not so recently (6 months ago or earlier) were less likely to quit than those who made a more recent (in last 6 months) quit attempt (OR = 0.23).

**Table 4:** Odds of making an attempt to quit smoking by Wave 2 (weighted; *n* = 1643)

		Wald χ^2^ Test		
	OR (95 % CI)	χ^2a^	DF	p value
Area				
Dhaka	1.00	11.98	1	<0.001
Areas outside Dhaka	3.41 (1.70, 6.82)			
Sex				
Male	1.00	2.82	1	0.093
Female	2.24 (0.87, 5.73)			
Age group				
Younger than 40	1.00	9.61	1	0.002
40 or older	1.53 (1.17, 2.00)			
Education				
Illiterate	1.00	1.69	2	0.429
1 to 8 years	1.08 (0.67, 1.75)			
9+ years	1.22 (0.89, 1.68)			
Income				
<5,000 BDT	1.00	5.37	3	0.147
5,000 to 10,000 BDT	1.11 (0.72, 1.73)			
>10,000 BDT	1.57 (1.07, 2.30)			
Not reported	1.14 (0.57, 2.25)			
Self-rated health				
Poor/average health	1.00	2.74	1	0.098
Good/excellent health	0.78 (0.59, 1.05)			
Intentions to quit				
No plans to quit	1.00	14.86	2	<0.001
Sometime in the future	1.73 (1.28, 2.32)			
Within the next 6 months	1.32 (0.76, 2.28)			
Advice to quit				
No advice	1.00	0.18	1	0.669
Advice/info/referral to quit	1.11 (0.70, 1.75)			
Workplace smoking restrictions				
No restrictions	1.00	9.10	2	0.011
Partial/complete restrictions	0.87 (0.56, 1.36)			
Does not work indoors/outside home	0.62 (0.43, 0.90)			
Home smoking restrictions				
No restrictions	1.00	4.97	2	0.083
Partial restrictions	0.92 (0.59, 1.43)			
Completely banned	1.74 (0.95, 3.19)			

*BDT* = Bangladeshi Taka; 1US$ = 79BDT

^a^Omnibus test

**Table 5:** Odds of successful cessation (6 months or longer) by Wave 2 (weighted; *n* = 454)

		Wald χ^2^ Test		
	OR (95 % CI)	χ^2^ ^a^	DF	p value
Area				
Dhaka	1.00	2.76	1	0.097
Areas outside Dhaka	2.09 (0.88, 4.99)			
Sex				
Male	1.00	0.10	1	0.747
Female	0.60 (0.03, 13.01)			
Age group				
Younger than 40	1.00	13.75	1	<0.001
Aged 40+	3.11 (1.71, 5.67)			
Education				
Illiterate	1.00	0.53	2	0.768
1 to 8 years	1.01 (0.48, 2.13)			
9+ years	1.27 (0.61, 2.68)			
Income				
<5,000 BDT	1.00	4.39	3	0.222
5,000 to 10,000 BDT	0.35 (0.12, 1.04)			
>10,000 BDT	0.39 (0.14, 1.05)			
Not reported	0.44 (0.09, 2.11)			
Quit recency				
Recently (in last 6 months)	1.00	11.29	2	0.004
Never tried to quit	0.75 (0.34, 1.66)			
Not so recently (6 months ago or earlier)	0.23 (0.09, 0.62)			
Self-rated health				
Poor/average health	1.00	7.91	1	0.005
Good/excellent health	2.40 (1.30, 4.43)			
Self-efficacy				
1 unit increase	1.75 (1.29, 2.38)	13.06	1	<0.001
Information/advice/referral to quit smoking				
No advice to quit	1.00	2.87	1	0.091
Advice/info/referral to quit	2.27 (0.88, 5.85)			
Outcome expectancy				
1 unit increase	0.82 (0.56, 1.20)	1.05	1	0.306

*BDT* = Bangladeshi Taka; 1US$ = 79BDT

^a^Omnibus test

## Discussion

This is the first nationally representative survey to study predictors of smoking cessation behavior among Bangladeshi adults. Our findings suggest that several factors are associated with quitting attempts and successful quitting among adult smokers from Bangladesh. The proportion of Bangladeshis who successfully quit smoking (4.3 %) was almost identical to the natural quit rate among other populations, such as Chinese adult smokers (4.4 %) [15]. However, one-fifth of adult smokers made quit attempts in this study though they were not successful, underscoring the need for organized smoking cessation programs. Previous research has shown that making attempts to quit is a strong predictor of successful quitting [16].

Consistent with findings from previous research [17, 18], in this study, older (aged 40 or above) smokers were more likely to make quit attempts or quit successfully. This may be due to the fact that older smokers experienced more health problems and visited more healthcare professionals and received repeated advise to quit smoking, which encouraged them to make quit attempts or quit successfully [19, 20]. In a Hong Kong study, older smokers wanted to set an example by quitting smoking for the future young generation [21]. The findings that smokers from areas outside the capital city of Dhaka had made more quit attempts than smokers who were residing inside the city underscore the fact that the limited tobacco use prevention and cessation campaigns in the city are not reaching the target population to encourage them to try to quit. The high quit intention among residents living outside Dhaka may also suggest the low level of income among these residents that limits their ability to buy cigarettes and, hence, they may make greater efforts to quit.

In this study, higher income smokers (monthly income above BDT 10,000) made more successful quit attempts compared to those with a monthly income of less than BDT 5,000. This suggests the need to target low income smokers with cessation interventions. In a study by Haas et al. [22], a proactive smoking cessation outreach program was effective in reaching low income smokers with smoking cessation services.

Although in previous studies home [23] or workplace [24] smoking restrictions had a significant effect on quitting attempts or quitting, the effect of these variables was not significant in the current study. In this study, respondents not working indoors/outside the home were less likely to have made a quit attempt than those with no workplace restrictions on smoking. This difference in our findings may have been due to the wide acceptance of smoking in Bangladeshi families and the dominating nature of smokers in the family who are mostly male. This phenomenon underscores the lack of enforcement of smoking restrictions in the home or workplace which might allow smokers to continue to smoke without affecting their quitting intentions. In a cross-sectional study of a large national sample of U.S. residents, Farkas et al. found that living in smoke-free households and working in smokefree workplaces had significant impacts on cessation [24].

The finding that recent quitting efforts (tried to quit less than six months ago) were associated with increased quitting success compared to making an attempt six months ago or earlier, is of note. These findings are most likely due to differences in smokers’ readiness to quit, while recent quitters try again and smokers without a recent quit attempt may simply not be ready to quit currently and thus not try to quit. This suggests the need to encourage smokers to make frequent quit attempts that should also reinforce their confidence to quitting, regardless of perceived likelihood of success on that quit attempt. In a study by Borland et al. [14] study, motivational factors predicted quit attempts but not maintenance of smoking cessation.

Consistent with the findings from other studies [25], perceiving one’s health as good or excellent was positively associated with quit success. This is due to the fact that those who are in good perceived health status practice more healthy behaviors and possess greater perceived benefits of quitting smoking. This group of people is more aware of the health risks of smoking and has high perceived vulnerability [26], which might mediate their quitting success. This underscores the need to incorporate risk perception as a construct within the population-based smoking cessation intervention programs.

The study has several limitations. First, our analysis was limited to daily smokers only, therefore, the findings may not be applicable to non-daily smokers. However, according to Wave 1 data, 96.1 % of cigarette smokers/dual users smoke cigarettes daily and 94.4 % of dual users smoke bidis daily, thus our results are representative for the vast majority of smokers in Bangladesh. Second, we limited successful quitters to those who quit for more than 6 months at the time of data collection, but some people will relapse [27], and thus our analysis probably included some people who did not quit permanently. Third, we were not able to examine the impact of using pharmacological therapy (i.e. nicotine replacement therapy or medications), if any, because the information was unavailable in the data set. Studies have reported that the use of such therapy predict quitting [28]. However, given the fact that these products were not registered and unavailable in Bangladesh at the time of the study, the probability of using these products by the respondents was very low. Fourth, because of the self-reported nature of smoking status, smoking could be underreported due to the respondents’ wish to give a socially desirable response [29, 30]. However, there is evidence that self-reports and biochemical measurements of serum cotinine concentration provide similar estimates of smoking prevalence in the United States [31]. Fifth, most of the earlier literature reported nicotine dependence as a predictor for smoking cessation [19, 20, 32]. However, we were unable to include nicotine dependence as a predictor of quitting attempts or smoking cessation due to the way data was collected in Wave 1. We did try to compensate for this by using CPD (total number of cigarettes smoked per day) and perceived addiction as predictors, although they were dropped from the model. It might be the case that a single measure such as nicotine dependence would have been better in this case. Finally, data were collected by trained interviewers who followed written interviewer guidelines. Any difference between their understanding and explanation of the questions asked could result in bias in information collected. However, such bias was minimized by the periodical observation of interviews by the senior research team members and investigator’s bi-weekly meetings with the interviewers.

In conclusion, the present study identified several predictors of quitting attempts and successful quitting amongst adult Bangladeshi smokers. The promoters of smoking cessation services should consider these factors when designing comprehensive tobacco control initiatives and in service planning. This could be done by identifying smokers who are not making any quitting attempt or were more likely to be unsuccessful in their quitting attempt, and then promoting targeted interventions for these groups of smokers. Our results call for an integrated intervention approach to promote smoking cessation at the population level which should target both social environmental and individual level factors. Efforts to intervene in quitting behavior (i.e. attempt to quit and smoking cessation) will have limited effectiveness unless they take into account the social environmental context in which such behavior takes place.

## Abbreviations

ITC: International Tobacco Control Policy Evaluation Project
BDT: Bangladeshi Taka
WHO: World Health Organization
AIC: Akaike information criterion
USDHHS: U.S. Department of Health and Human Services
